# Pennsylvania Hospital

**Published:** 1850-01

**Authors:** James J. Levick

**Affiliations:** Resident Physician, Pennsylvania Hospital


PENNSYLVANIA HOSPITAL.—*Surgical Wards.—Service of* DR. NORRIS.

*Cases discharged from November 1st, to December 1st*, 1849.

| | Cured. | By request. | Died. |
|---|---|---|---|
| Abscess, | 1 | 0 | 0 |
| Burns, | 1 | 0 | 1 |
| Caries | 1 | 0 | 0 |
| Concussion of brain, | 0 | 0 | 1* |
| Conjunctivitis, | 1 | 0 | 0 |
| Contusion, | 2 | 0 | 0 |
| Diseased bladder, | 1 | 0 | 0 |
| Dislocation of elbow, | 1 | 0 | 0 |
| Fractures 27, simple 22, viz.: | | | |
| Arm, | 1 | 0 | 0 |
| Calcis, | 1 | 0 | 0 |
| Clavicle, | 1 | 0 | 0 |
| Forearm, | 7 | 0 | 0 |
| Hand, | 1 | 0 | 0 |
| Jaw, lower, | 1 | 0 | 0 |
| Leg, | 3 | 0 | 0 |
| Patella, | 1 | 0 | 0 |
| Rib, | 1 | 0 | 0 |
| Skull, | 0 | 0 | 2† |
| Thigh, | 2 | 1‡ | 0 |
| Fractures, compound, 5 viz.: | | | |
| Finger, | 1 | 0 | 0 |
| Foot, | 1‖ | 0 | 0 |
| Leg, | 2 | 0 | 0 |
| Skull, | 1 | 0 | 0 |
| Gonorrhœa, | 2 | 0 | 0 |
| Inflamed hand, | 4 | 0 | 0 |
| Do. knee, | 1 | 0 | 0 |
| Paronychia, | 3 | 0 | 0 |
| Retention of urine, | 1 | 0 | 0 |
| Scrofulous disease of testis, | 0 | 1 | 0 |
| Swelled testicle, | 1 | 2 | 0 |
| Sprain, | 1 | 0 | 0 |
| Syphilis, | 5 | 0 | 0 |
| Ulcers, | 1 | 0 | 0 |
| Wounds 11, viz.: | | | |
| Gunshot, | 4 | 0 | 0 |
| Incised, | 2 | 1 | 0 |
| Lacerated, | 4 | 0 | 0 |
| Punctured, | 1 | 0 | 0 |
| | 62 | 5 | 4 |

* Of abscess of the brain.

‡ Of neck of femur.

† One of abscess of brain.

‖ By amputation.

A case of dislocation backwards of both bones of the forearm, without fracture, very similar to that reported in the May number of the Examiner, 1849, has been discharged within a week or two. The patient, a lad 12 years of age, was suddenly pushed over a carpenter's bench, striking his elbow as he fell. He was immediately brought to the hospital. The luxation was very evident, but after a close examination no fracture could be detected. As in the case above alluded to, the olecranon was found some distance above its usual position, the forearm was semi-flexed, the patient unable to bend the joint and complaining of pain when any attempt was made to extend the forearm, while anteriorly the lower end of the humerus could be felt apparently projecting over the joint.

From pain and fright the boy had become nauseated, the muscles were thus much relaxed, so that reduction was readily effected by placing the knee at the elbow joint, making gentle traction at the wrist, and by bending the forearm. The bones glided into their place with a snap and the deformity was at once corrected. A roller commencing at the hand was carried up the arm, and an angular splint applied to its anterior surface. Little or no inflammation followed the injury. In a few days the splint was laid aside, slight motion of the joint made, a sling substituted, and in eight days the boy went out with full motion of the articulation.

As above stated, the lad believed his elbow to have been struck as he fell; this may have been the case, though it is not improbable that in falling, the hand was put forward to protect himself, and the injury thus received, the luxation being, it is said, sometimes produced in this manner.

Three fatal cases of fracture of the skull are reported. The first of these was that of an Irishman aged 32 years, whose head was crushed by two rail road cars, which he was stooping to detach, having been suddenly forced together. He was brought to the hospital in a dying condition, and expired in about an hour. The appearance presented by his head was somewhat peculiar. There was no external wound communicating with the skull, the fracture being a simple one, but a distinct ridge of bone could be felt along the whole length of the parietal and part of the frontal bones, these having evidently yielded at their sutures, an opinion confirmed by a post-mortem examination. By this was ascertained the existence of a comminuted fracture of the temporal bone of the left side and a complete overlapping of the parietal bones at their mesial line.

In the second instance, the man had fallen from the upper story of a high building, striking a ladder as he fell, by which the violence of the fall was somewhat broken. When brought to the hospital he was laboring under symptoms of concussion of the brain. His head was severely bruised, his left eye much swelled and ecchymosed, but there was no external wound, no paralysis and no insensibility to light. Hemmorhage from the nose, and it was believed a slight bleeding from the ear, took place at the time of the accident.

Cold to his head, warmth to his feet, perfect rest, the use of mercurials, occasional cupping and strict attention to his diet, constituted his treatment, under which he improved rapidly. On the eighth day after his injury he appeared decidedly convalescent, his intellect perfect, and free from pain in the head. During the afternoon he imprudently ate some cakes and candies brought by his friends, and in the night was attacked with severe pain in the head and stomach followed by nausea and vomiting. These symptoms continued throughout the night and in the morning the patient was found slightly convulsed, and with increasing dullness of intellect. His bowels were freely moved by cathartics and enemata, cups were applied to his head, and a large blister to the nape of the neck. He continued to grow worse, involuntary discharges of feces, total loss of his faculties, paralysis and frequent convulsions followed, and the man died two days subsequently.

Much to our regret, permission to examine the body was positively refused.

In this case it is possible that the crude food taken, may have been an immediate exciting cause of cerebral disturbance, in a brain already diseased, though there was little doubt of a fracture at the base of the cranium, and that an abscess of the brain had been insidiously forming.

In another, somewhat similar case, that of an Irishman, aged 28 years, who had fallen through a hatchway, on the side of his head, the symptoms of injury at the base of the brain were almost unequivocal from the beginning. When brought to the hospital he was found bleeding from the nose and ears, and with entire loss of intellect. Partial paralysis was subsequently detected. His treatment was in the main similar to that above mentioned, varied to meet particular indications. A gradual improvement followed, the paralysis somewhat abated, his intellect improved, though he never fully recovered the possession of his faculties. A few days before his death, he complained of severe pain in his head, and a sero-purulent discharge from his right ear took place. One or two paroxysms of noisy delirium, and an increase of paralysis, followed, and the man died on the 29th ult., a fortnight after the accident. His death was somewhat sudden, he having been seen but a few minutes before, apparently no worse than on the preceding day. A post-mortem examination of his head was made, by which was found a fracture of the squamous portion of the right temporal bone and a large coagulum pressing directly on the brain. This last having been removed, a well marked impression was left on the substance of the brain. On removing the cerebral mass, a fracture of the petrous portion of the temporal bone was discovered. At the base of the brain, at a point immediately above the fracture, as if caused by the irritation of the rough edges of the broken bone, was found a large abscess, extending to the lateral ventricle, and full of fetid pus.

An Irish labourer, aged 48 years, entered the hospital on the 14th of September, having fallen on his feet from a ladder ten or twelve feet high, fracturing his left os calcis. There was but little deformity, but he complained of pain when the foot was moved, and the two fragments of bone could be distinctly felt grating against each other, upon slight lateral motion.

The leg was placed in a fracture box, and the foot supported by the foot board. Considerable tenderness and swelling followed, which abated under the application of cooling lotions. Union took place slowly, but satisfactorily. At the end of five weeks the box was laid aside, and a figure of eight bandage applied over the instep and heel, in such a manner as to aid in retaining the uniting fragments, and a week later the patient was permitted to rise. Several days elapsed before he could bear any weight on the foot, but by the aid of crutches, he so improved as to be able to go out, walking without difficulty, about eight weeks after the receipt of the injury.

This accident is spoken of by all surgical authorities as one of rare occurrence; the experience of the hospital coincides with this statement, no other case of this fracture having been treated here for a long time. South, in his edition of Chelius' Surgery, quotes two or three cases of this accident. In one of these the posterior part of the bone was drawn up to a considerable extent, by the action of the gastrocnemii muscles. I find a somewhat similar case reported in the American Journal of Medical Sciences, for 1829, (page 129). In this the bone " was fractured just below the tendo achillis, and the posterior portion was drawn up as high as five inches from its former position." The patient recovered, though with deformity, the displacement of the bone never having been overcome. A case very like this was, I believe, under the care of Dr. J. Rhea Barton, several years since. Londsdale, in his treatise on fractures, has described a very ingenious apparatus for treating this accident, especially when deformity exists. In the case above described, the injury to the soft parts was less than usually happens, and the posterior fragment was not drawn upward, being no doubt retained in its place by the inferior calcaneo-cuboid ligament. The fracture was oblique, and a little posterior to the middle of the bone. No other treatment was here required than rest, the fracture box and a roller.

The four patients with gun-shot wounds, referred to in the last report, have since that time been discharged, all of them apparently in good health. JAMES J. LEVICK, Resident Physician.

*Pennsylvania Hospital December*, 1849.

